# Neuronal RNA‐binding protein dysfunction in multiple sclerosis cortex

**DOI:** 10.1002/acn3.51103

**Published:** 2020-07-01

**Authors:** Hannah E. Salapa, Catherine Hutchinson, Bogdan F. Popescu, Michael C. Levin

**Affiliations:** ^1^ Department of Anatomy, Physiology and Pharmacology & Cameco MS Neuroscience Research Center College of Medicine University of Saskatchewan Saskatoon SK Canada; ^2^ Office of the Saskatchewan Multiple Sclerosis Clinical Research Chair University of Saskatchewan Saskatoon SK Canada; ^3^ Neurology Division Department of Medicine University of Saskatchewan Saskatoon SK Canada

## Abstract

**Objective:**

Neurodegeneration is thought to be the primary cause of neurological disability in multiple sclerosis (MS). Dysfunctional RNA‐binding proteins (RBPs) including their mislocalization from nucleus to cytoplasm, stress granule formation, and altered RNA metabolism have been found to underlie neurodegeneration in amyotrophic lateral sclerosis and frontotemporal dementia. Yet, little is known about the role of dysfunctional RBPs in the pathogenesis of neurodegeneration in MS. As a follow‐up to our seminal finding of altered RBP function in a single case of MS, we posited that there would be evidence of RBP dysfunction in cortical neurons in MS.

**Methods:**

Cortical neurons from 12 MS and six control cases were analyzed by immunohistochemistry for heterogeneous nuclear ribonucleoprotein A1 (hnRNP A1) and TAR‐DNA‐binding protein‐43 (TDP‐43). Seven distinct neuronal phenotypes were identified based on the nucleocytoplasmic staining of these RBPs. Statistical analyses were performed by analyzing each phenotype in relation to MS versus controls.

**Results:**

Analyses revealed a continuum of hnRNP A1 and TDP‐43 nucleocytoplasmic staining was found in cortical neurons, from neurons with entirely nuclear staining with little cytoplasmic staining in contrast to those with complete nuclear depletion of RBPs concurrent with robust cytoplasmic staining. The neuronal phenotypes that showed the most nucleocytoplasmic mislocalization of hnRNP A1 and TDP‐43 statistically distinguished MS from control cases (*P* < 0.01, *P* < 0.001, respectively).

**Interpretation:**

The discovery of hnRNP A1 and TDP‐43 nucleocytoplasmic mislocalization in neurons in MS brain demonstrate that dysfunctional RBPs may play a role in neurodegeneration in MS, as they do in other neurological diseases.

## Introduction

Multiple sclerosis (MS) is a demyelinating autoimmune disorder of the central nervous system (CNS) in which neurodegeneration plays a significant role in its pathogenesis. One of the key pathological features of MS is the development of demyelinated lesions or plaques in the CNS. Plaques, depending on their stage, can contain numerous immune cells, including T cells and macrophages. In addition to inflammation, lesions also show evidence of axonal injury.^1^ Multiple mechanisms underlying neurodegeneration in MS have been proposed, including axonal transport deficits, mitochondrial dysfunction, and autoantibodies to nonmyelin antigens. Recently, we have provided evidence from a single MS case that a novel mechanism may underlie neuronal damage in MS.^2^ We have shown neuronal mislocalization of the RNA‐binding protein (RBP) heterogeneous nuclear ribonucleoprotein A1 (hnRNP A1) from its homeostatic nuclear location to the cytoplasm, where it forms aggregates. Additionally, we have found that these hnRNP A1 cytoplasmic accumulations colocalize with a marker of stress granules.

RBPs are essential in maintaining RNA homeostasis within a cell and play roles in mRNA stability, function, and transport. In addition to MS, dysfunctional RBPs, including hnRNP A1 and TAR‐DNA‐binding protein‐43 (TDP‐43), have been implicated in neurological disorders such as amyotrophic lateral sclerosis (ALS), Alzheimer's disease, and frontotemporal dementia (FTLD).^3^, ^4^, ^5^, ^6^, ^7^, ^8^, ^9^ Common features of dysfunctional RBPs in these diseases include mislocalization of the RBP from its homeostatic nuclear location to the cytoplasm and the formation of protein aggregates in the cytoplasm of cells. We and others have shown that dysfunctional RBPs, including hnRNP A1 and TDP‐43, are prominent pathological features in relevant animal models of MS.^10^, ^11^ We demonstrated that TDP‐43 and hnRNP A1 mislocalization are prevalent in spinal cord neurons of animals with experimental autoimmune encephalomyelitis. Additional analyses revealed that hnRNP A1 mislocalization correlated with disease severity, CD3^+^ immune cell infiltrates, SMI‐32 staining, a marker of neurodegeneration, and neuronal cell death.^10^

Given these data suggesting that dysfunctional RBP biology may contribute to neurodegeneration in a model of MS and our previous observations in human tissue, we sought to determine whether hnRNP A1 and TDP‐43 mislocalization are features of neurons in MS cortex. We have found an increased incidence of differential distribution of the RBPs hnRNP A1 and TDP‐43 in normal appearing cortical neurons from 12 MS cases as compared to controls. Specifically, we found that MS cases displayed a significantly higher proportion of neurons with nuclear depletion and cytoplasmic accumulation of hnRNP A1 and TDP‐43. Assessment of these phenotypic changes in hnRNP A1 and TDP‐43 distribution within neurons from MS cortices, as compared to controls, suggests that dysfunctional RBPs may be involved in MS pathogenesis in a manner similar to other neurological diseases.

## Materials and Methods

### Cases and autopsy material

Study approval was granted by the University of Saskatchewan Biomedical Research Ethics Board (Bio#11‐217 and Bio#17‐207). Formalin‐fixed paraffin‐embedded autopsy material from 12 MS cases and six control cases with no known neurological deficits was used (Table 1). Clinical details were available for many, but not all, cases.

**Table 1:** Patient demographic and clinical characteristics of non‐MS controls and neuropathologically confirmed MS cases.

Sample ID	Age	Sex	Clinical subtype	Symptom onset (year)	Postmortem interval (h)	Diagnosis (year)
NL1	26	M	Control	N/A	N/A	N/A
NL2	41	M	Control	N/A	16	N/A
NL3	N/A	N/A	Control	N/A	N/A	N/A
NL4	76	M	Control	N/A	N/A	N/A
NL5	74	F	Control	N/A	N/A	N/A
NL12	N/A	N/A	Control	N/A	N/A	N/A
MS8	48	M	MS	N/A	N/A	N/A
MS9	54	M	MS	N/A	N/A	N/A
MS10	58	M	SPMS	31	N/A	1
MS11	56	F	PPMS	14	N/A	7
MS12	44	M	RRMS	1	N/A	1
MS13	57	F	MS	N/A	3	25
MS14	44	M	PPMS	N/A	17	15
MS15	44	F	MS	N/A	9	N/A
MS16	65	F	MS	N/A	2	N/A
MS17	55	M	SPMS	N/A	3	N/A
MS22	N/A	F	SPMS	N/A	N/A	N/A
MS23	49	F	SPMS	27	7.5	21

Clinical data, including age, sex, clinical subtype, symptom onset and diagnosis, were included for each sample, if available. The postmortem interval was also noted, if available. NL, control; MS, multiple sclerosis; M, male; F, female; SPMS, secondary progressive MS; PPMS, primary progressive MS; RRMS, relapsing remitting MS; N/A, not available/applicable.

### Immunohistochemistry

Tissue was sectioned at 5 µm and mounted onto slides before undergoing deparaffinization and rehydration through a xylene and ethanol gradient. Endogenous peroxidases were blocked using 0.2% hydrogen peroxide in methanol. Heat‐mediated antigen retrieval was performed by placing slides in 10 mmol/L Tris/Ethylenediamine tetraacetic acid (EDTA) buffer for 45 min in a steamer. Sections were blocked in 10% fetal bovine serum (FBS) in phosphate buffered saline (PBS) for 15 min before being incubated with primary antibodies diluted in the same solution overnight at 4°C. The following primary antibodies were used: rabbit anti‐TDP‐43 (Novus Biologicals, Centennial, CO) and mouse anti‐hnRNP A1 (Millipore, Burlington, MA). The next day, sections were washed and incubated with the appropriate biotinylated secondary antibody for 1 h at room temperature and then incubated in avidin peroxidase for 1 h at room temperature. Slides were developed using 3,3′‐diaminobenzidine and counterstained with hematoxylin.

### Immunofluorescence

Tissue was sectioned, deparaffinized, and subjected to antigen retrieval as above. Following antigen retrieval, slides were washed in PBS before being placed into PBS + 0.3% Triton X‐100 for 20 min at room temperature for permeabilization. Slides were washed and treated with TrueBlack (Biotium, Fremont, CA) for 2 min as per the manufacturer's protocol. Sections were blocked for 1 h at room temperature in 10% FBS in PBS before being incubated with primary antibody diluted in 5% FBS in PBS overnight at 4°C. The same primary antibodies were used as above. Following overnight incubations, slides were washed and incubated with donkey anti‐mouse AlexaFluor 488 and donkey anti‐rabbit AlexaFluor 594 (both from Jackson Immunoresearch, West Grove, PA) secondary antibodies diluted in 5% FBS in PBS for 1 h at room temperature. Sections were washed and further incubated with rabbit anti‐NeuN conjugated AlexaFluor 647 (Abcam, Cambridge, UK) for 1 h at room temperature before being counterstained with 4′,6‐diamidino‐2‐phenylindole (DAPI) and mounted with ProLong Gold mounting medium.

### Quantitative analysis

**Figure 1:**
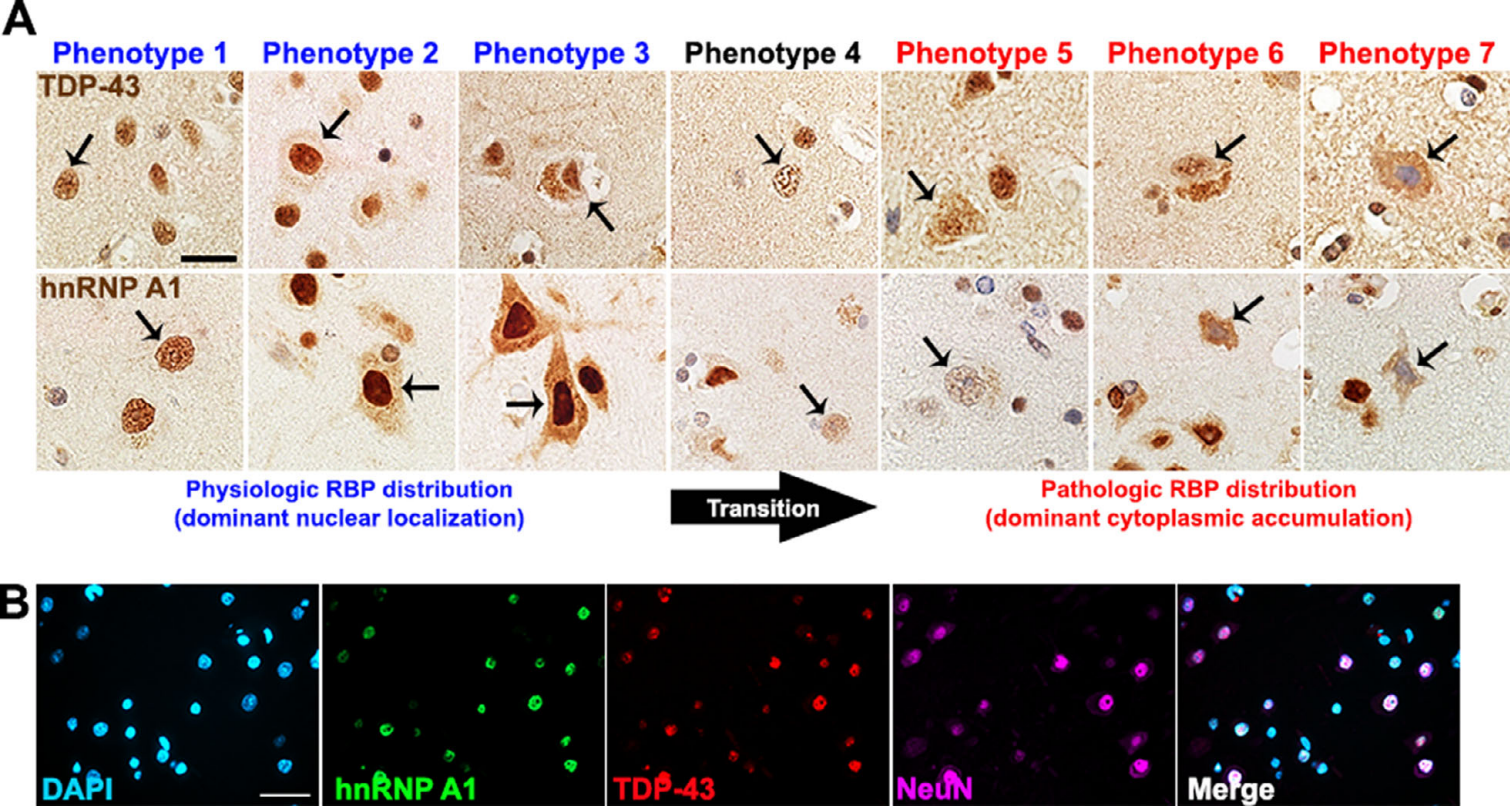
TDP‐43 and hnRNP A1 phenotypes observed in human neurons. (A) Representative images of the seven different RBP phenotypes with TDP‐43 and hnRNP A1 staining in cortical neurons. Arrows indicate neurons that illustrate the phenotype being described in the corresponding row. Phenotypes 1–3 are associated with physiological localization of hnRNP and TDP‐43 where the RBP demonstrates primarily nuclear localization. Conversely, phenotypes 5‐7 have been associated with pathologic RBP distribution where there is decreased nuclear staining and dominant cytoplasmic accumulation of a RBP. There may be a transition stage (arrow, transition) where neurons exhibit decreased RBP nuclear localization but not cytoplasmic accumulation. Scale bar 20 μm. (B) Immunofluorescence images demonstrate phenotype 1 distribution of hnRNP A1 (green) and TDP‐43 (red) in NeuN^+^ neurons (pink). Scale bar 50μm. TDP‐43, TAR‐DNA‐binding protein‐43; RBP, RNA‐binding protein; hnRNP A1, heterogeneous nuclear ribonucleoprotein A1.

A block from each control and MS case was randomly selected for quantitative analyses. Images of normal appearing cortex were taken at 40× magnification using an Olympus BX53 microscope equipped with an Olympus DP72 camera (Olympus Canada Inc., Ontario, Canada). Approximately 20 images for normal appearing cortex were acquired per case. Images were imported into ImageJ and individual neurons were assessed for the phenotype of the RBP of interest. Examiners were not blinded when assessing the images. Seven phenotypes were defined in order to assess RBP distribution in each neuron (Table 2, Fig. 1), ranging from a physiological, normal appearing phenotype (Phenotype 1: robust nuclear RBP staining, no cytoplasmic staining) to a more severe mislocalization phenotype associated with pathological RBP distribution (Phenotype 7: nuclear depletion of RBP, cytoplasmic accumulation of RBP). Groups of at least 150 neurons were counted across the acquired images for each individual case and percentages were determined by dividing the number of positive neurons for a phenotype by the total number of neurons counted. This yielded a phenotype distribution plot for each individual case representing the variation in the RBP phenotypes present.

**Table 2:** RNA‐binding protein phenotype scoring system for quantification.

Phenotype no.	Description
1	Robust, nuclear RBP staining, no cytoplasmic staining
2	Robust, nuclear RBP staining, faint cytoplasmic staining
3	Robust, nuclear RBP staining, robust cytoplasmic staining
4	Faint or sparse nuclear RBP staining, no cytoplasmic staining
5	Faint or sparse nuclear RBP staining, faint cytoplasmic staining
6	Faint or sparse nuclear RBP staining, robust cytoplasmic staining
7	Nuclear depletion of RBP, cytoplasmic staining

RBP, RNA‐binding protein.

### Statistical analysis

Statistical analyses were performed using GraphPad Prism 5 software. For comparisons between individual phenotypes in control and MS normal appearing cortex, one‐tailed unpaired tests were used to determine differences. *P* < 0.05 was considered statistically significant.

## Results

### Normal appearing cortex from MS patients exhibited differential distribution of TDP‐43 phenotypes as compared to controls

**Figure 2:**
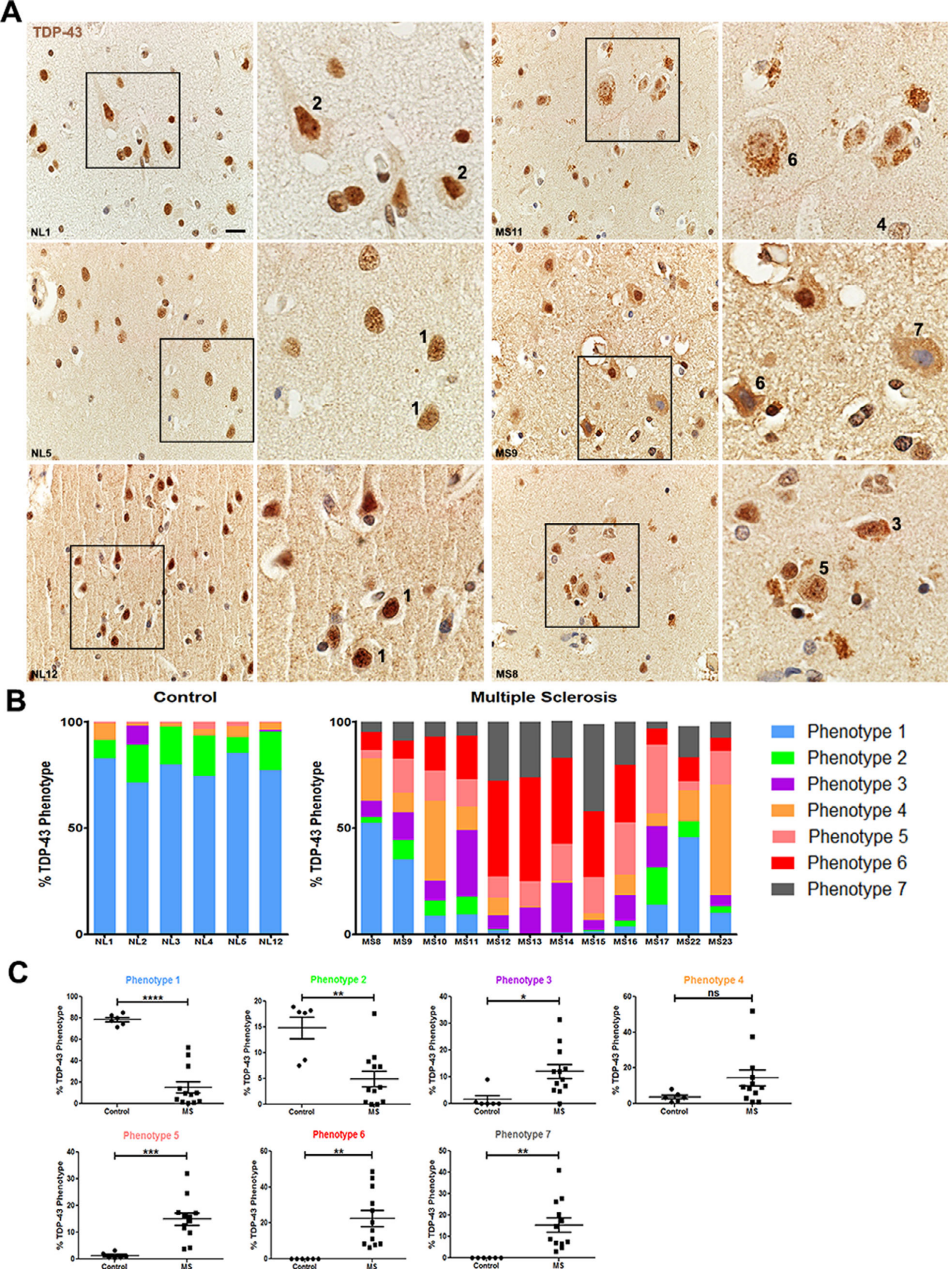
Differential distribution of TDP‐43 phenotypes in control and MS normal appearing cortex. (A) Representative images of TDP‐43 staining in neurons from cortex from three different control cases and three different MS cases. Nuclei are counterstained with hematoxylin. Numbers refer to the phenotypes defined and illustrated in Table 2 and Figure 1. Scale bar 20μm. (B) Quantification and distribution of the seven different RBP phenotypes when examining TDP‐43 staining in neurons from cortex for each control case (*n* = 6) and MS case (*n* = 12). For analyses, images were taken from normal appearing cortex. (C) Graphical illustration of the differences between control and MS cases for each individual TDP‐43 phenotype. Data are graphed as the percentage of neurons positive for that phenotype where the denominator is the number of neurons counted in that case (150–340 neurons per case). Each circle (controls, *n* = 6) or square (MS, *n* = 12) represents a value from an individual case. Unpaired t‐test for statistical analysis. *P < 0.05, **P < 0.01, ***P < 0.001, ****P < 0.0001. Error bars represent standard error of mean. TDP‐43, TAR‐DNA‐binding protein‐43; RBP, RNA‐binding protein; NL, control, MS, multiple sclerosis; n.s., not significant.

Previous reports of TDP‐43 mislocalization in neurons from other neurologic diseases, demonstrated severe phenotypes where TDP‐43 shifts from being mainly nuclear to almost exclusively cytoplasmic under disease conditions.^12^, ^13^ Interestingly, in vitro experiments have described a gradient of TDP‐43 mislocalization in which TDP‐43 transitions from being exclusively nuclear to being nuclear with faint cytoplasmic staining to faintly nuclear and robustly cytoplasmic.^8^, ^14^, ^15^ In order to account for the varying degrees of mislocalization, we described seven neuronal RBP phenotypes to stage TDP‐43 localization in this study (Table 2, Fig. 1). Neurons from control cases (*n* = 6) predominantly exhibited phenotypes 1 and 2, accounting for approximately 80–90% of quantified neurons, characterized by robust TDP‐43 staining of the nucleus (Fig. 2B). Varying numbers of neurons, depending on the control case, exhibited phenotype 4, with TDP‐43 faintly staining the nucleus. In controls, no neurons were found to be phenotypes 6 or 7, where TDP‐43 exhibits a pattern of nuclear depletion and cytoplasmic mislocalization. Conversely, in normal appearing cortex of MS patients, the percentage of neurons exhibiting phenotypes 1 and 2 decreased to varying degrees across all cases but no cases exhibited more than 60% of these nuclear TDP‐43 phenotypes (Fig. 2B). The appearance of phenotypes 6 and 7 in normal appearing cortex neurons, in which TDP‐43 was minimal in the nucleus and accumulates within the cytoplasm, was robust in the majority of the examined MS cases. This suggests a marked shift in neuronal TDP‐43 phenotype from mostly nuclear TDP‐43 localization in controls to an increased cytoplasmic accumulation in MS cases (Fig. 2A). Individual TDP‐43 phenotypes were compared between control and MS cases. A significant reduction in the number of neurons exhibiting phenotypes 1, 2, and 3 was found in MS cases as compared to controls (Fig. 2C, *P* < 0.0001, *P* < 0.01, *P* < 0.05, respectively). There was a significant increase in the number of neurons positive for phenotypes 5, 6, and 7 in MS cases as compared to controls (Fig. 2C, *P* < 0.001 for all).

### Differential distribution of hnRNP A1 phenotypes in normal appearing cortex from MS patients

**Figure 3:**
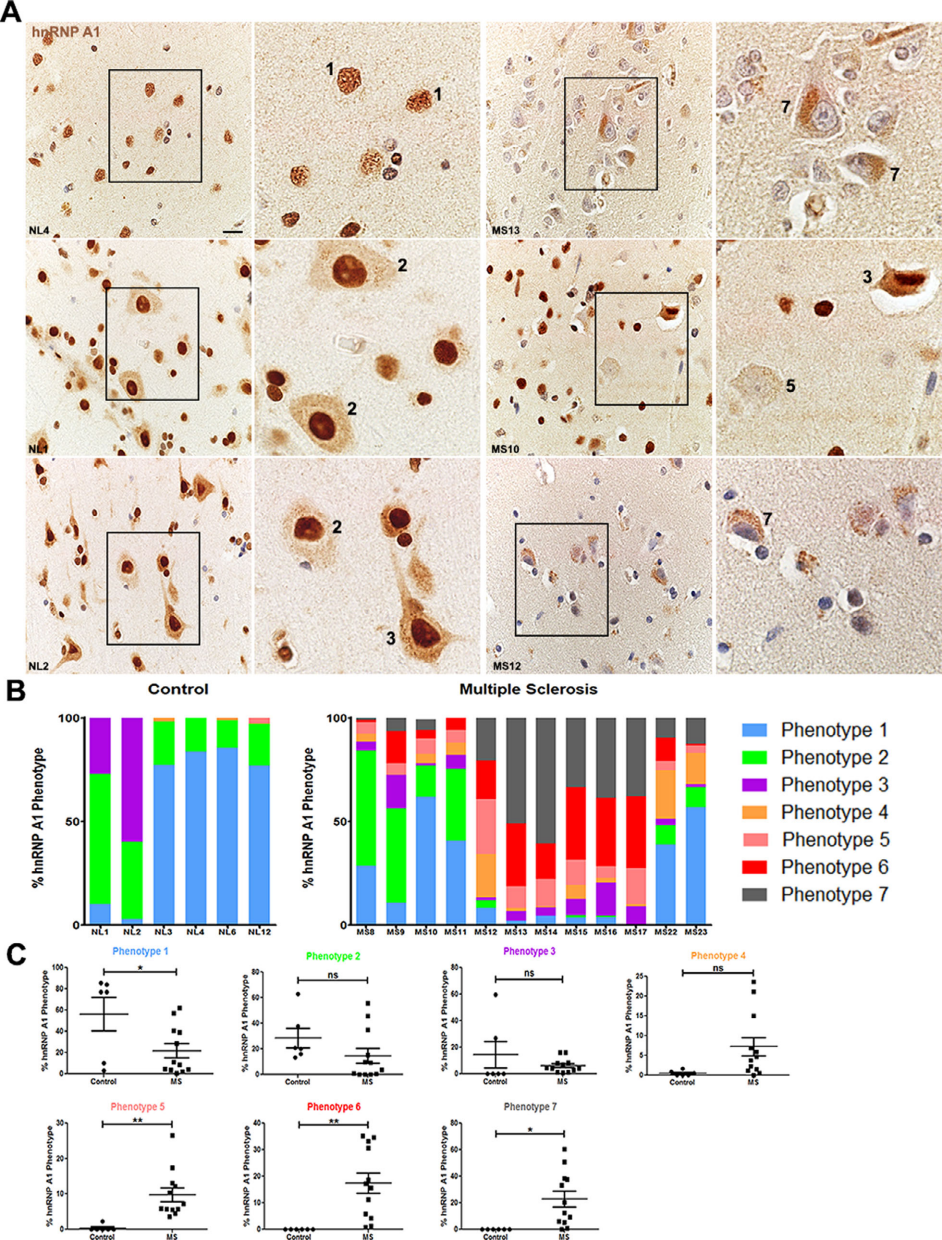
Differential distribution of hnRNP A1 phenotypes in control and MS normal appearing cortex. (A) Representative images of hnRNP A1 staining in neurons from cortex from three different control cases and three different MS cases. Nuclei are counterstained with hematoxylin. Numbers refer to the phenotypes defined and illustrated in Table 2 and Figure 1. Scale bar 20μm. (B) Quantification and distribution of the seven different RBP phenotypes when examining hnRNP A1 staining neurons from cortex from each control case (*n* = 6) and MS case (*n* = 12). For analyses, images were taken from normal appearing cortex. (C) Graphical illustration of the differences between control and MS cases for each individual hnRNP A1 phenotype. Data are graphed as the percentage of neurons positive for that phenotype where the denominator is the number of neurons counted in that case (165–280 neurons per case). Each circle (controls, *n* = 6) or square (MS, *n* = 12) represents a value from an individual case. Unpaired t‐test for statistical analysis. *P < 0.05, **P < 0.01. Error bars represent standard error of mean. *NL* control, *MS* multiple sclerosis, n.s. not significant. MS, multiple sclerosis; hnRNP A1, heterogeneous nuclear ribonucleoprotein A1.

We previously published a single case of MS exhibiting hnRNP A1 nuclear depletion of cortical neurons.^2^ As with TDP‐43 distribution, hnRNP A1 may vary in its cellular distribution based on cell type and condition.^16^ In order to account for the gradient of hnRNP A1 distribution patterns, we used the same seven neuronal RBP phenotypes as described above to phenotypically assess hnRNP A1 localization in our study (Table 2, Fig. 1). Neurons from control cases predominantly showed phenotypes 1 and 2 of nuclear hnRNP A1 localization, accounting for approximately 50–80% of the neurons that were quantified (Fig. 3). However, two of the control cases displayed an increased prevalence of phenotype 3 with robust hnRNP A1 staining of both the nucleus and cytoplasm. Three control cases contained neurons which displayed less than 5% of phenotypes 4 or 5. Furthermore, no control cases were found to contain neurons exhibiting phenotypes 6 or 7 (Fig. 3). In the normal appearing cortex of MS patients, the presence of neurons positive for phenotypes 1 and 2 decreased (Fig. 3B) suggesting a reduction in nuclear hnRNP A1 localization. Furthermore, all of the MS cases examined contained hnRNP A1 phenotypes 6 and 7 in normal appearing cortex neurons, suggesting a marked shift from nuclear to cytoplasmic hnRNP A1 distribution as compared to control cases (Fig. 3C). Several cases robustly replicated our seminal findings of neuronal hnRNP A1 nuclear depletion (Fig. 3B). We further performed individual comparisons of each hnRNP A1 phenotype to determine differences between control and MS cases. MS cases displayed a significant reduction in phenotype 1 as compared to control cases (Fig. 3C, *P* < 0.05). Phenotypes 2, 3, and 4 were found to not be statistically different between control and MS cases. However, MS cases contained significantly more normal appearing cortex neurons exhibiting phenotypes 5, 6, and 7 as compared to controls (Fig. 3C, *P* < 0.01 for all). These data closely parallel the phenotypic distribution changes we also observed during the TDP‐43 phenotype analyses.

### Mislocalization of hnRNP A1 and TDP‐43 within the same neurons in MS normal appearing cortex

**Figure 4:**
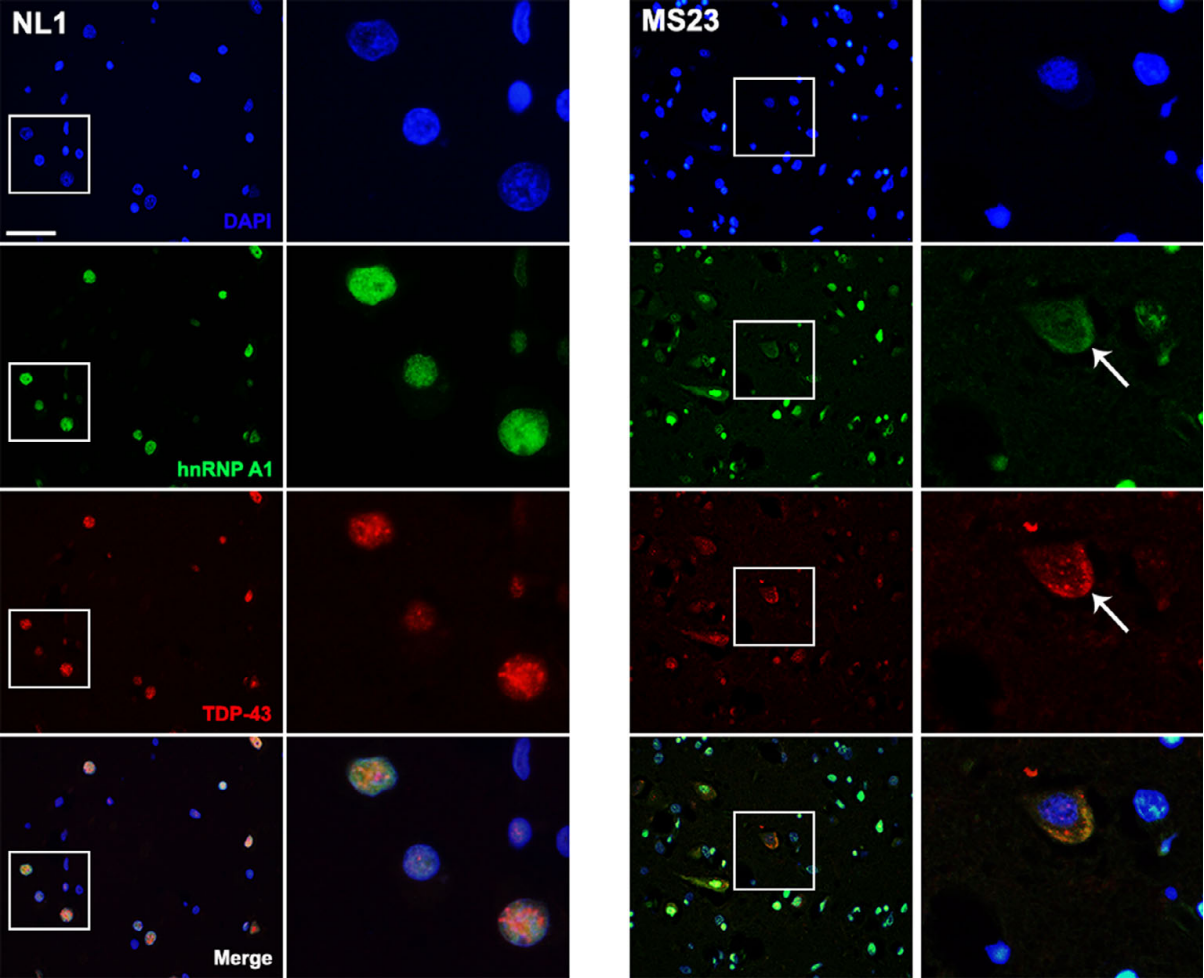
Mislocalization of hnRNP A1 and TDP‐43 within the same neurons in MS normal appearing cortex. Immunofluorescence shows colocalization of hnRNP A1 and TDP‐43 in the cytoplasm of neurons from MS brain. Control brain (NL1) shows nuclear (DAPI‐blue) localization of hnRNP A1 (green) and TDP‐43 (red) and colocalization in the nucleus (merged image). A representative neuron from MS (MS23) shows that there is decreased nuclear staining and cytoplasmic accumulation of hnRNP A1 and TDP‐43 (arrow) suggesting colocalization of mislocalized hnRNP A1 and TDP‐43 within the same neuron. Scale bar 50μm. hnRNP A1, heterogeneous nuclear ribonucleoprotein A1; TDP‐43, TAR‐DNA‐binding protein‐43; MS, multiple sclerosis.

Considering that we observed similarities in the phenotypic distribution changes between TDP‐43 and hnRNP A1 in MS cases, we performed immunofluorescence for both RBPs in a control and MS case to determine if there was colocalization of TDP‐43 and hnRNP A1 in neurons. Neurons from control normal appearing cortex demonstrated predominantly nuclear colocalization of TDP‐43 and hnRNP A1, illustrative of phenotype 1 (Fig. 4). In contrast with controls, MS normal appearing cortex showed decreased nuclear staining of hnRNP A1 and TDP‐43 and increased, robust cytoplasmic mislocalization within the same neuron (Fig. 4), some of which colocalized. Both hnRNP A1 and TDP‐43 demonstrated phenotype 6 RBP distribution within this neuron suggestive of a relationship between the two RBPs.

## Discussion

We previously published data from a single MS case compared to a non‐MS control demonstrating nuclear depletion of hnRNP A1, an RBP important in mRNA splicing and transport.^2^ We have expanded these findings from a single case to multiple MS cases and included an additional RBP, TDP‐43. The heterogeneity of TDP‐43 phenotypes in FTLD is well studied.^4^, ^5^, ^17^ Abnormal TDP‐43 structures are typically classified into neuronal cytoplasmic inclusions, neuronal preinclusions, neuronal intranuclear inclusions, and dystrophic neurites and the distribution of and presence of these TDP‐43 features has been used to subtype FTLD and AD cases.^18^, ^19^, ^20^ Consistent brain regions are affected by FTLD and AD and can be more readily assessed for TDP‐43 pathology. MS is a heterogeneous disease that affects different parts of the CNS in different cases and to varying severities. In order to account for the amount of heterogeneity observed in MS and the varying degrees of RBP mislocalization reported in the literature,^8^, ^14^ we used a method of quantifying and phenotypically classifying RBP localization in tissue. These phenotypes ranged from homeostatic RBP nuclear localization to mild mislocalization and to severe RBP nuclear depletion with cytoplasmic accumulation, which are features of pathologic neurons in other neurologic diseases.

Using this phenotypic classification system, we analyzed 12 MS cases and six control cases compromising of more than 2700 cortical neurons for hnRNP A1 and TDP‐43 localization. We found that both of these RBPs exhibited differential phenotypic distribution in neurons in the normal appearing cortex of MS patients as compared to controls. Specifically, MS patients showed a reduction in the number of neurons with nuclear hnRNP A1 and TDP‐43 and a significant increase in the number of neurons with decreased nuclear localization and cytoplasmic mislocalization. Cortical neuron phenotypes 6 and 7 have the greatest degree of cytoplasmic staining (representative of nucleocytoplasmic mislocalization), which has been strongly associated with neurodegeneration and neuronal injury in other neurological diseases and their models.^10^, ^14^, ^21^, ^22^, ^23^, ^24^ Remarkably, we could not find any of phenotypes 6 or 7 in cortical neurons of normal control brain, while there were at least some of these neuronal phenotypes in every MS case studied.

Nuclear localization of RBPs is believed to be physiologic while varying degrees of mislocalization have been shown to be detrimental to cellular processes and cell health and associated with neurologic disease pathology. In ALS, there is a correlation with the degree of mislocalization and disease severity in that increased amounts of cytoplasmic RBPs are associated with more severe forms of disease.^25^, ^26^ Several hypotheses have been proposed to explain the pathological and damaging effects of RBP mislocalization. The first hypothesis is a gain of toxicity in the cytoplasm postulating that increased cytoplasmic concentrations of an RBP alters its liquid‐liquid phase separation dynamics leading to the formation of insoluble protein aggregates.^27^, ^28^ Decreased RNA availability in the cytoplasm can cause RBPs to become more aggregate prone^27^ while increasing concentrations of proteins that have the potential to aggregate increases the probability of liquid to solid aggregate transitions.^28^ Furthermore, in ALS mouse models, TDP‐43 has been shown to mislocalize to the cytoplasm and abnormally bind and splice cytosolic RNA targets.^26^, ^29^ The presence of insoluble aggregates in a cell might create problems with the protein clearance machinery negatively impacting cellular health. Interestingly, our results demonstrate colocalization of hnRNP A1 and TDP‐43 to the cytoplasm of neurons in MS patients but not controls, suggesting that the formation of RBP aggregates although further experiments are needed to confirm this.

Other researchers suggest that dysfunctional RBPs lead to neurodegeneration through loss of function of the RBP in the nucleus. Nuclear perturbations of a target RBP suggest it is no longer performing its necessary functions within the nucleus. For example, nuclear depletion of TDP‐43 leads to lack of proper RNA processing, including disrupted splicing regulation, transcription, control, and micro‐RNA processing^30^ and has been shown to be sufficient to induce neuronal cell death.^23^ Findings from others often demonstrate nuclear depletion or loss of nuclear RBPs in conjunction with cytoplasmic accumulation of the RBP in neurons suggesting that it is most likely a combination of RBP gain of toxicity in the cytoplasm and loss of function in the nucleus that contributes to neuronal dysfunction and death.^26^ Our data suggest that a similar mechanism may be involved in MS pathogenesis whereby hnRNP A1 and TDP‐43 nuclear depletion and cytoplasmic accumulation lead to neuronal injury through gain of toxicity in the cytoplasm and loss of nuclear function. Further experiments are needed to elucidate the precise mechanism by which hnRNP A1 and TDP‐43 dysfunction influence neuronal health in MS.

A number of factors, including oxidative stress, proinflammatory cytokines, and inflammation, have been shown to influence RBPs in neurons.^2^, ^31^, ^32^, ^33^ Furthermore, mutations within RBPs, including hnRNP A1 and TDP‐43, have been shown to be causative of ALS and FTLD. We have previously shown that proinflammatory cytokines, antibodies to hnRNP A1, and MS‐associated mutations within hnRNP A1 lead to its mislocalization from nucleus to cytoplasm in in vitro models.^2^, ^34^, ^35^ We and others have also demonstrated that hnRNP A1 and TDP‐43 neuronal mislocalization is a prominent feature in mouse models of MS.^11^, ^36^ Particularly, we found that the degree of hnRNP A1 mislocalization positively correlated with experimental autoimmune encephalomyelitis disease severity and negatively correlated with neuronal cell count.^36^ This suggests that the severity of RBP mislocalization may be related to neurodegeneration and disease progression. Although it is unclear what is influencing RBP distribution in MS and related rodent models, it is clear that dysfunctional RBP biology is a prominent feature and could be influencing disease progression and pathology in a manner similar to other neurologic diseases.

This study was limited to RBP distribution in cortical neurons. However, research from others indicates that RBP localization may also be important in oligodendrocytes.^11^, ^37^ For example, selective depletion of TDP‐43 in oligodendrocytes in mice leads to increased mortality, decreased myelination, and neurological deficit development.^37^ These experiments are further supported by data demonstrating RBP mislocalization, specifically TDP‐43, in oligodendrocytes in the Theiler's murine encephalomyelitis virus model of MS.^11^ The role of RBP dysfunction in other glial cells, such as microglia and astrocytes, which are particularly relevant to MS pathology, is relatively unclear although research suggests that inflammation can induce RBP mislocalization in these cell types as well.^31^ Furthermore, recent proteomic analyses determined that TDP‐43 was differentially regulated in T cells from MS patients as compared to healthy controls further suggesting a role for dysregulated TDP‐43 and other RBPs in MS pathology.^38^

Overall, we believe that this robust analysis of TDP‐43 and hnRNP A1 localization in cortical neurons in MS patients demonstrates a role for dysfunctional RBP biology in disease and suggests a novel mechanism underlying neurodegeneration in MS.

## Author Contributions

HES contributed experimental data and analyses and wrote and edited the manuscript. CH contributed the experimental data. BFP contributed human pathology expertise. MCL contributed to the experimental design and interpretation, reviewed, edited, and approved the final version of the manuscript, including figures. All authors read and approved the final manuscript.

## Conflicts of interest

The authors declare no conflicts of interest.
